# Characteristics of Factors Influencing the Occurrence of Cleft Lip and/or Palate: A Case Analysis and Literature Review

**DOI:** 10.3390/children11040399

**Published:** 2024-03-28

**Authors:** Małgorzata Kulesa-Mrowiecka, Anna Lipowicz, Bożena Anna Marszałek-Kruk, Damian Kania, Wojciech Wolański, Andrzej Myśliwiec, Krzysztof Dowgierd

**Affiliations:** 1 Department of Rehabilitation in Internal Diseases, Institute of Physiotherapy, Faculty of Health Sciences, Jagiellonian University Medical College, 31-126 Krakow, Poland; m.kulesa-mrowiecka@uj.edu.pl; 2 Department of Anthropology, Institute of Environmental Biology, Wroclaw University of Environmental and Life Sciences, 50-375 Wroclaw, Poland; anna.lipowicz@upwr.edu.pl; 3 Department of Genetics, Wroclaw University of Environmental and Life Sciences, 51-631 Wroclaw, Poland; bozena.marszalek-kruk@upwr.edu.pl; 4 Laboratory of Physiotherapy and Physioprevention, Institute of Physiotherapy and Health Sciences, Academy of Physical Education, 40-065 Katowice, Poland; d.kania@awf.katowice.pl (D.K.); a.mysliwiec@awf.katowice.pl (A.M.); 5 Department of Biomechatronics, Faculty of Biomedical Engineering, Silesian University of Technology, 41-800 Zabrze, Poland; wojciech.wolanski@polsl.pl; 6 Head and Neck Surgery Clinic for Children and Young Adults, Department of Clinical Pediatrics, University of Warmia and Mazury, 10-561 Olsztyn, Poland

**Keywords:** cleft, pediatric cleft lip palate, ethiology

## Abstract

Introduction: Cleft lip with or without cleft palate (CL/P) stands as the most common congenital facial anomaly, stemming from multifactorial causes. Objective: Our study aimed to ascertain the prevalence and characteristics of cleft palates, identify associated risk factors to inform prevention and prenatal detection for early intervention, and assess postoperative rehabilitation protocols for cleft palates. Design: This study employs a retrospective descriptive and clinical approach. Patients: The study includes 103 children with cleft palates treated at the Department of Head and Neck Surgery Clinic for Children and Young Adults, Department of Clinical Pediatrics, University of Warmia and Mazury. Methods: We conducted a thorough evaluation of records, considering variables such as sex, cleft type, maternal occupation, parental education, and family history of clefts. Data analysis was carried out using R software version GPL-3 and ordinal logistic regression analyses. Results: Notably, children born to mothers who experienced significant stress during pregnancy exhibited a 9.4-fold increase in the odds of having bilateral cleft palates. Conversely, no substantial evidence was found to support the influence of the child’s sex, birth order, body mass, maternal exposure to workplace toxins, infections, or drug toxicity on the dependent variable. Conclusions: Our findings suggest that children with parents who have a history of clefts and those with less educated mothers are more likely to develop bilateral cleft palates. Additionally, children born to mothers experiencing stress during pregnancy face an increased risk of bilateral cleft palates. It is important to note that there is a paucity of literature on rehabilitation following various cleft palate surgical techniques in children.

## 1. Introduction

Our study addresses the critical global health issue of cleft lip and/or palate (CL/P), focusing on understanding its complex causes to improve healthcare outcomes. By analyzing individual cases and existing research, we aim to identify contributing factors to CL/P, facilitating the development of more effective prevention and intervention strategies. CL/P, resulting from genetic, environmental, and teratogenic influences, requires a comprehensive understanding of effective management and genetic counseling. Our findings distinguish between syndromic and isolated clefts, with syndromic clefts associated with genetic disorders and isolated clefts arising from a mix of factors. This differentiation helps in tailoring clinical management and underlines the need for ongoing research to explore CL/P’s multifaceted nature further [1]. 

Isolated clefts make up about 70% of cleft lip and palate (CL/P) cases and 50% of cleft palate (CP) cases, influenced by both internal and external factors [2,3]. The incidence of clefts varies globally, being more common among Asians and Native Americans (1 in 500) and less common in African populations (1 in 2500) [4]. CL/P is the most prevalent congenital facial anomaly worldwide, with a rate of 7.94 per 10,000 live births [5]. In Poland, it is one of the leading congenital defects, occurring in about 10 per 10,000 births [6].

Cleft conditions exhibit a gender-based prevalence, more commonly occurring in males than females. The distribution is as follows: cleft lip is found in 59.5% of males compared to 40.5% of females; cleft palate shows a prevalence of 57.62% in males and 38.58% in females; and for cases involving both cleft lip and palate, 42.38% are male, and 29.52% are female. Specifically, isolated cleft lip (without palate involvement) is significantly more common in males (61.12%) than females (2.52%). Additionally, cleft lip predominantly affects the left side and about half of the patients with a cleft lip also present a cleft palate, indicating a fusion issue in facial development before palate formation [7].

Cleft lip and palate occurrences are observed in 50% of patients, often resulting from the improper fusion of facial structures before palate formation. The causes are categorized into non-genetic factors, such as environmental influences (e.g., smoking, alcohol consumption), and genetic factors, which include clefts associated with other malformations or appearing as isolated incidents [8]. Various genes have been identified with mutations that can cause clefts, showing a connection between the type of mutation and the resulting phenotype. Treatment is age-specific and requires timely intervention, involving a multidisciplinary team including otolaryngologists, geneticists, speech pathologists, and orthodontists. Most research points to a blend of genetic and environmental factors behind cleft formation, with multiple genes identified for non-syndromic cleft lip and palate. Additionally, recent studies suggest different etiological factors for the three types of clefts (lip, palate, lip, and palate), although the precise molecular mechanisms are yet to be fully understood (Table 1 and Table 2). Research has also highlighted the role of DNA methylation patterns during embryonic development in influencing cleft occurrences, with analyses of blood samples from affected children revealing both known and new gene sequences associated with various methylation patterns [9,10,11].

The etiology of cleft lip and palate involves both genetic and environmental factors, with recent findings supporting different causes for its three subtypes: cleft lip, cleft palate, and combined cleft lip and palate. Scientific and epidemiological research highlights the significant impact of environmental risk factors, including smoking, alcohol consumption, malnutrition during pregnancy, viral infections, teratogenic drugs, folate deficiency, and body mass, on the development of these congenital anomalies. These environmental influences, alongside genetic predispositions, contribute to the complex embryonic development leading to cleft formation [12,13,14].

Children with cleft lip and palate face challenges in speech, feeding, and hearing, as well as cosmetic and psychological issues, necessitating comprehensive and long-term multidisciplinary care. They also exhibit higher mortality rates up to the age of 55 and an increased risk of cancer. The embryological development of the face involves the frontonasal process and the maxillary and mandibular processes. Clefts arise from disruptions in the fusion of these processes between the 4th and 12th weeks of fetal life, with cleft lip resulting from failures in the fusion of the maxillary process and nasal processes, and cleft palate from the failure of palatal processes to fuse [15].

The LAHSHAL Cleft Classification system is a detailed method for classifying cleft phenotypes, based on anatomical structures from right to left: Right lip, Right alveolus, Right hard palate, Soft palate midline, Left hard palate, Left alveolus, and Left lip. This system, capable of describing over 12,000 anatomical and severity combinations of cleft conditions, is valued for its specificity but is limited by its complexity [16].

### 1.1. Aim of Study

The study aims to improve the management of cleft palate by identifying genetic and environmental risk factors, enhancing early intervention, and developing personalized care strategies. It focuses on a comprehensive evaluation of patients’ and families’ backgrounds, including ethnicity, socio-economic status, and genetic history, to tailor prevention and treatment approaches. The research also emphasizes the importance of understanding educational levels and age-specific needs to create effective communication and educational materials. Gender-specific studies will explore differences in cleft palate outcomes, guiding personalized treatment plans. The study intends to advance genetic research and rehabilitation methods, ensuring treatments are tailored to each child’s unique genetic makeup. Additionally, it prioritizes innovative postoperative rehabilitation and robust support systems for families, addressing their mental, emotional, and financial challenges. The goal is to not only treat cleft palate but also enhance the long-term health and quality of life of affected children, fostering their societal integration. Collaborations with healthcare providers, educators, and community organizations will ensure a holistic treatment and support approach, making care for children with cleft palate more effective and personalized. Data and data preparation

Participants were recruited during their hospital visit at the Department of Head and Neck Surgery Clinic for Children and Young Adults, Department of Clinical Pediatrics, University of Warmia and Mazury, Olsztyn. The appropriate consent/assent forms were collected. The potential participants, patient representatives, were also contacted remotely by telephone to introduce the retrospective study. Once eligible participants provide verbal consent to participate, the researcher either collects data face-to-face or remotely. The study included children whose parents or legal caregivers, after reading the aim, scope, and course of the study, provided written informed consent to conduct study procedures and to process the personal data of the child in accordance with the Ordinance of the European Parliament and of the Council of EU of 27 April 2016, on the protection of individuals. The study was approved by the Ethics Committee of the Academy of Physical Education, Katowice, Poland; (ID KBE-1/2021/28/10/21; date of approval: 28 October 2021) and was conducted in accordance with the Declaration of Helsinki of the World Medical Association concerning ethical procedures for medical studies involving human participants.

The data collected in case report forms were entered into a secure electronic database. Descriptive statistics were used to summarize relevant data regarding frequency, risk factors, interventions, and status of children with cleft palate.

### 1.2. Materials

The initial sample of 103 children was reduced by 18 individuals due to missing data, which included 7 birth weights, 5 orders of a live child born, 2 histories of cleft in any of parents, 11 education levels of mother, and 1 cleft record. The remaining data includes 36 girls and 46 boys, 14 children being born third or higher in order, and 13 children having small or very small body mass (<2500 g).

### 1.3. Statistical Methods

The data were analyzed using R (R core team 2021). In this article, we focus on modeling a cleft whose level can be coded in many ways. Since arbitrarily different coding methods for this variable can theoretically lead to qualitatively different outcomes, we performed a type of sensitivity analysis using two models in which the dependent variable was either (A) an ordered tertiary classification or (B) a score. The first type of coding (A) includes 3 classes: midline or unilateral incomplete, unilateral complete, and bilateral. Table 3 shows the classification of cleft cases in correspondence to LASHSAL coding. The second model (B) is based on score, which is computed by counting the presence of cleft in any lip (L or l), alveolus (A or a), hard palate (H or h), soft palate (S or s), and uvula (U) as well as the presence of bilateral cleft. The score is standardized for better comparability with model (A), so that the midline or unilateral incomplete clefts fall in the range from 0 to 1, unilateral complete clefts have a value of 1, and bilateral clefts fall in the range from 1 to 2.

Since the first model (A) has an ordered categorical dependent variable, we use generalized ordinal logistic regression (VGAM R package, version GPL-3, Yee 2010 [5] This type of regression assumes.

That at least some of the variables violate the proportional odds assumption. The set of variables inconsistent with this assumption was determined using the AICc selection (The Akaike Information Criterion (AIC) is a method used in statistics to choose among multiple competing models, with the goal of selecting the model that best explains the data while penalizing for the number of parameters to avoid overfitting) [17] from among all possible subsets of the set of all independent variables. The selection of the AICc model revealed that only intercept, toxic risk at the mother’s work, and maternal stress violate the proportional odds assumption. The second model (B), which has points as the dependent variable, was fitted using the standard linear model in Table 4 and Table 5.

## 2. Results

**Figure 1:**
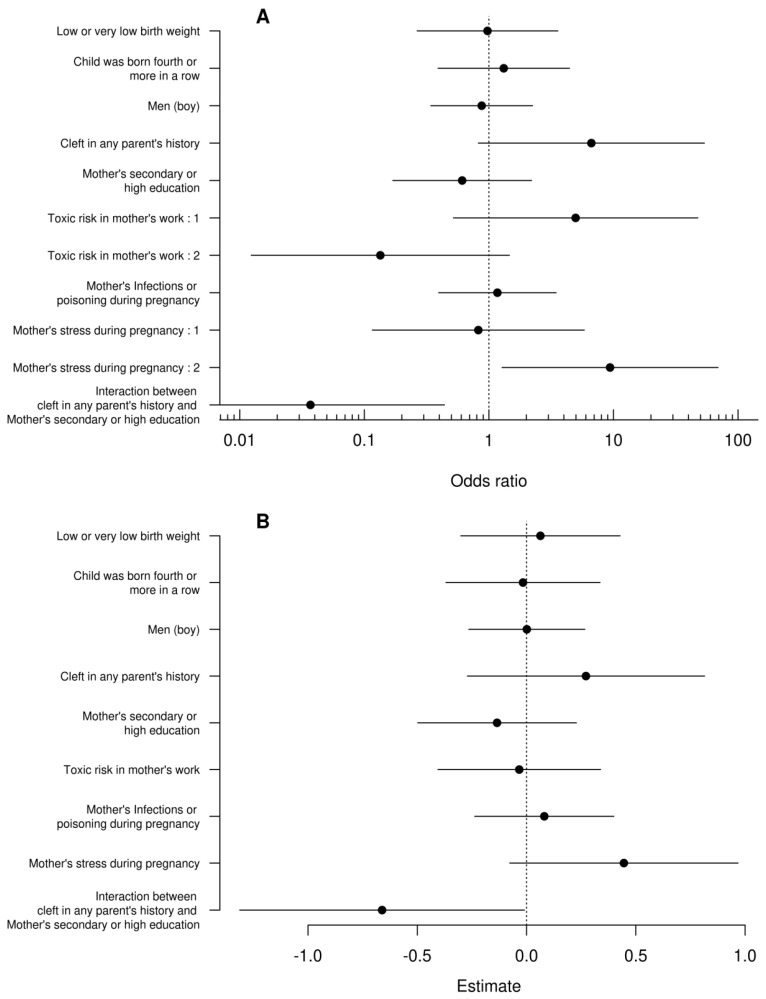
Confidence intervals of odds ratios for the generalized ordinal logistic regression (**A**) and standard linear regression coefficients (**B**). The vertical dashed lines denote no effects. Interpreting the interaction between the parent’s cleft story and the mother’s education can cause some problems with this graph so it is analyzed separately in Figure 2.

**Figure 2:**
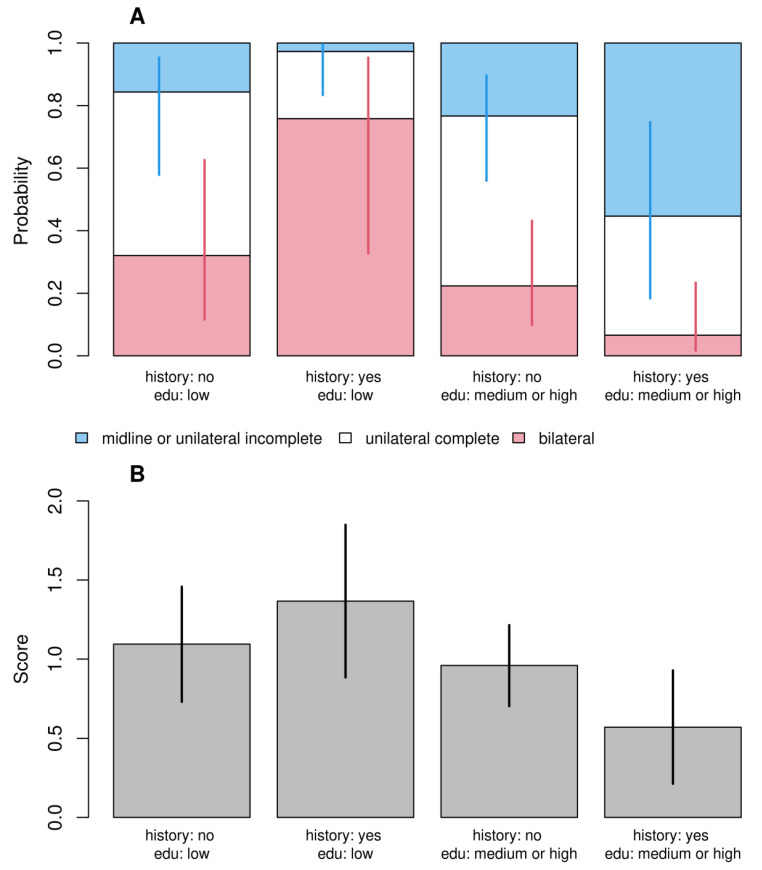
Predicted probabilities (**A**) and scores (**B**) for different combinations of parent’s cleft history and maternal education for the generalized ordinal logit (**A**) and standard linear (**B**) models. The figure illustrates the effect of the interaction between these two variables. The values of the remaining independent variables were set at their reference levels.

Despite very different modeling approaches, the results of both models were qualitatively consistent. In the first model (Figure 1A), we observed that children of stressed mothers during pregnancy had 9.4 times higher odds of having bilateral cleft. Similarly, in the second model (Figure 1B), these children obtained a score higher by half a point (1/4 of the maximum score value). While there was no evidence that children’s sex, order of birth, and body mass as well as risk of maternal toxicity at work or reporting infections or drug toxicity had a significant effect on the dependent variable, we found a statistically significant interaction between parents’ cleft history and maternal education in both models. Children of parents with cleft history and having low-educated mothers had a higher probability of bilateral cleft and lower probability of the simplest cleft (Figure 2A) as well as higher scores (Figure 2B) than children of parents with cleft history and having medium and highly educated mothers. Children of parents with a cleft history and having poorly educated mothers had a higher probability of bilateral cleft palate. The study in question meticulously investigates the complex influences on the occurrence of bilateral cleft palate in children, emphasizing the significant role of maternal stress during pregnancy. Utilizing a detailed questionnaire administered to a varied cohort of families, the research examines a spectrum of maternal factors—including genetic predisposition, educational attainment, occupational hazards, health risks during pregnancy, and particularly psychological stress—to assess their impacts on the development of craniofacial anomalies.

The methodology of the survey involved categorizing children based on distinct maternal backgrounds and yielded insightful quantitative data. It identified 24 children with a familial history of cleft conditions, highlighting a genetic factor. Additionally, 60 children were born to mothers with secondary or higher education levels, pointing to socio-economic influences on health outcomes. The survey also focused on occupational risks, with 12 children having mothers exposed to potential toxins in their workplaces. Such occupations included hairdressing and chemistry, flagged for their chemical exposures and classified as high-risk, contrasting with professions such as teaching and economics, deemed lower risk due to their minimal physical strain and hazardous material exposure.

Furthermore, the study reported on 18 children whose mothers experienced infections or drug toxicity during pregnancy, and notably, 6 children whose mothers reported significant stress. This maternal stress was quantitatively linked to a 9.4 times higher likelihood of offspring developing bilateral cleft palate compared to those not exposed to such stress, underlining the profound effect of psychological stressors on fetal development.

The definition of maternal stress in this context is comprehensive, encompassing any psychological, physiological, or environmental stressor that could negatively impact fetal development through hormonal changes or direct toxic effects. These stressors include, but are not limited to, emotional distress, financial or occupational pressures, health concerns, or exposure to harmful substances. This broad definition highlights the multifaceted nature of stress and its potential to affect pregnancy outcomes significantly.

This detailed exploration into the survey’s methodology and findings underscores the intricate web of factors contributing to the incidence of bilateral cleft palate and other craniofacial anomalies. The research advocates for a multidimensional approach to prenatal care and postnatal rehabilitation, highlighting the necessity to address not only the physical aspects of such conditions but also the broader environmental, socio-economic, and psychological factors that influence both occurrence and recovery.

## 3. Discussion

The study investigated the influence of genetics, socio-economic status, and environmental factors on the incidence of bilateral cleft palates in children, uncovering significant associations. Notably, maternal stress during pregnancy emerged as a critical risk factor, increasing the likelihood of bilateral clefts by 9.4 times. Additionally, the risk escalates with a combination of low maternal education and a family history of cleft conditions, indicating the importance of socio-economic factors. The research highlights a deficiency in comprehensive rehabilitation strategies, suggesting a need beyond conventional physiotherapy and speech therapy. It also identifies certain maternal occupations as higher risk due to potential toxic exposures, emphasizing the complex interplay of factors in craniofacial development and the necessity for enhanced care and prevention strategies.

Further findings include the impact of non-modifiable risk factors like gender and ethnicity on cleft occurrences, with males more commonly affected by cleft lip with or without palate, and females more by cleft palate alone. Ethnicity also plays a role, with higher cleft rates observed in Asians, whites, and indigenous populations compared to African populations, suggesting a protective factor in non-white groups [18,19].

Nutritional status during pregnancy, particularly a diet low in vitamin B12 and folate, is linked to a higher risk of clefts, underscoring the importance of a maternal diet. Additionally, certain foods, like those high in solanine, are associated with an increased risk of clefts and neural tube defects, pointing to the significance of dietary choices during pregnancy.

This study underlines the necessity for a multifaceted approach to understanding and addressing the risk factors of cleft palates, advocating for comprehensive care, improved prevention strategies, and consideration of the diverse factors affecting craniofacial development [20,21,22,23,24,25,26].

The study highlights stress as a critical environmental factor impacting fetal development, notably increasing the risk of cleft lip and palate [2,27,28].

Stress elevates maternal and fetal cortisol levels, known for their teratogenic effects, by downregulating the enzyme 11-beta-hydroxysteroid dehydrogenase type 2 (11beta HSD2), crucial for regulating the placental barrier. This process is particularly detrimental during early pregnancy stages, essential for facial formation. Elevated cortisol levels can lead to hyperinsulinemia and insulin resistance, further harming fetal development. However, the negative impacts of stress can be mitigated by vitamin B6 supplements, which act as suppressors of tissue receptors for corticosteroids.

Additionally, stress increases catecholamine levels, potentially reducing uterine blood flow and increasing the risk of fetal oxygen deprivation. Research suggests a correlation between maternal psychological stress during the 15th week of pregnancy and cleft lip and palate incidence. The study also examined the role of maternal physical activity, finding a weak association with cleft occurrence, with prolonged sitting emerging as a potential protective factor, though further research is needed to understand this relationship fully. In summary, the study underscores the complex interplay of stress, nutrition, and physical activity in the risk of developing cleft lip and palate, advocating for comprehensive care and preventive strategies during pregnancy to mitigate these risks [29,30].

Furthermore, deficiencies in vital nutrients such as folate (vitamin B9) and cobalamin (vitamin B12) play a significant role in fetal development. These deficiencies, resulting from poor dietary intake or genetic mutations affecting the folate cycle, are critical for DNA synthesis and repair, emphasizing the importance of balanced diets and supplementation during pregnancy. The enzyme MTHFR, essential for converting folic acid into its active form, supports cell differentiation and tissue growth during embryogenesis [31].

Thus, folic acid and cobalamin supplementation during pregnancy is recommended to reduce the risk of clefts and neural tube defects, despite uncertainties regarding the relationship between clefts and folate pathway polymorphisms [32].

Vitamins, including folic acid, are commonly prescribed during pregnancy, with a recommended intake of about 400 mcg of folic acid daily from conception through the first 12 weeks to decrease the risk of neural tube defects and clefts. The absence of vitamin supplementation is linked with an increased risk of cleft lip and palate, highlighting the genetic and embryological bases of these conditions [33,34,35,36]. 

Nutritional deficiencies, especially in folate and vitamin B12, along with certain maternal medications like antiepileptics and painkillers, have been flagged for their potential to increase cleft risks. The study stresses the importance of balanced diets and careful medication management during pregnancy to mitigate these risks. Additionally, it notes that antibiotics, particularly beta-lactams, sulfonamides, and macrolides, must be chosen carefully due to their debated links to cleft formation.

Diabetes, characterized by elevated blood glucose levels, manifests in various forms: Type 1 (insulin-dependent), Type 2 (insulin-resistant), and gestational diabetes occurring during pregnancy. The worldwide diabetes prevalence stands at 8.3%, with gestational diabetes, particularly alongside obesity, tripling the risk of fetal congenital anomalies like clefts. This underscores the critical need for stringent blood glucose control in pregnant women to prevent such defects. Moreover, the use of antihypertensive medications in early pregnancy presents teratogenic risks, necessitating cautious management to avoid congenital abnormalities, including clefts [37,38].

Infections during early pregnancy can negatively impact lip and palate development, increasing cleft risks. Thus, preventive actions against infections are essential for protecting the fetus from such congenital defects. Specifically, hyperthermia from viral infections significantly raises the likelihood of clefts, advising pregnant individuals to avoid high fever conditions. Early pregnancy infections associated with cleft risks include colds, flu, appendicitis, and urinary and genital infections, highlighting the importance of prompt treatment and vaccinations, such as the flu shot, to safeguard fetal health [39,40,41,42,43].

Additionally, there is no evidence linking SARS-CoV-2 infection during pregnancy to an increased risk of clefts. However, maternal illnesses like anemia during pregnancy might relate to cleft conditions, potentially due to embryonic hypoxia affecting facial formation in the first trimester. The role of inflammatory cytokines is also considered in these developmental issues [44].

The risk of cleft palate may elevate with maternal thyroid hyperactivity during pregnancy. The use of painkillers and antiepileptic medications, necessary for managing conditions like gallstones and neuro-musculoskeletal pain, must be carefully evaluated for their teratogenic potential. Pregnant individuals and healthcare providers should meticulously balance medication benefits against fetal development risks to make informed decisions minimizing potential adverse effects [45].

The third trimester has been associated with an increased risk of cleft palate and cleft lip and palate, underscoring the critical need for careful selection and timing of antibiotic use during pregnancy. The debate over the safety of antibiotic use highlights the complexity of ensuring maternal health while protecting fetal development. Healthcare providers must weigh the benefits of treating infections against potential risks to the fetus, with particular attention to the timing of exposure and the specific type of antibiotic prescribed. This careful consideration is essential to minimize the risk of congenital anomalies such as cleft lip and palate, ensuring both maternal and fetal health are optimally managed [46,47,48].

Corticosteroids’ teratogenic potential calls for cautious use, especially in early pregnancy, to avoid increasing cleft formation risks. Various other medications, including antiepileptic drugs, antidepressants, and opioids, are associated with a higher incidence of clefts, highlighting the critical need for healthcare providers to balance treatment efficacy with fetal safety [49,50,51].

The research underscores the complex interplay of factors contributing to the risk of cleft lip and palate, encompassing genetics, environmental influences, and lifestyle choices. Diabetes, particularly when paired with obesity, triples the risk of fetal congenital defects, including clefts, due to hyperglycemia’s adverse effects. The study also highlights the teratogenic risks associated with the early pregnancy use of antihypertensive medications and the adverse impact of infections and hyperthermia on lip and palate development.

The importance of careful medication management during pregnancy is underscored by the association between the use of certain medications and an increased risk of cleft lip and palate. Medications such as antiepileptic drugs, retinoic acid, painkillers, benzodiazepines, antidepressants, stimulants, antihypertensive drugs, and even those containing iron and folic acid, require careful consideration due to their potential teratogenic effects. Healthcare providers must evaluate the necessity of these medications against their potential risks, tailoring treatment plans to each patient’s unique circumstances to minimize harm to the developing fetus [52,53,54,55,56,57,58].

Furthermore, lifestyle factors and substance misuse significantly impact fetal development. Opioid misuse during pregnancy not only triples the risk of cleft lip and palate but also increases the likelihood of Neonatal Abstinence Syndrome (NAS) in newborns. Similarly, smoking and alcohol consumption during early pregnancy are identified as significant risk factors for cleft formation, with the severity of the risk increasing with the dose of alcohol consumed. These findings highlight the critical need for comprehensive prenatal care and education, emphasizing the avoidance of harmful substances and the cautious use of medications to prevent congenital defects such as cleft lip and palate. Substance misuse, including opioid use and smoking, significantly elevates cleft risks, as does alcohol consumption during the first trimester. The study advocates for enhanced prenatal care education and comprehensive approaches to prevention and treatment, considering the myriad factors influencing cleft lip and palate risk without significant contributions from gender or maternal occupation. This holistic perspective is vital for developing effective strategies to reduce the incidence of clefts and ensure better health outcomes for affected individuals [59].

Understanding the complexities of treating bilateral cleft lip andpalate requires a comprehensive analysis that encompasses the comparison of surgical protocols, the implications of patients changing hospitals or clinical protocols, and the role of genetic factors in treatment outcomes. Surgical protocols vary significantly in terms of techniques, timing, and the provision of pre- and post-surgical care, and rehabilitation services. As highlighted by Mossey et al. [60] the diversity in surgical approaches underscores the need for clinicians and families to understand these differences to contextualize patient outcomes within the broader landscape of cleft lip and palate treatment. The timing of surgery [61], plays a critical role in determining the effectiveness and long-term results of the treatment, making it imperative to evaluate the rationale behind surgical schedules.

The continuity of care is another critical aspect, particularly when patients move to another town or switch healthcare providers, leading to changes in the clinical protocol. Author [61] emphasize the challenges and potential disruptions in treatment continuity that can significantly affect patient outcomes. This underscores the importance of examining the reasons behind such changes and their impact on the care and recovery of patients with orofacial clefts.

Furthermore, the role of genetics in orofacial clefts has become a vital area of research. Genetic testing can provide valuable insights into the success or complications of surgical interventions. Beaty et al. [62] have demonstrated the importance of identifying genetic markers associated with cleft lip and palate, highlighting the correlation between genetic factors and surgical outcomes. This evolving understanding of genetics emphasizes the need for incorporating genetic testing findings into the treatment protocol to enhance the personalization and effectiveness of care for patients [63].

By weaving these critical elements into the discussion, a study can offer a more nuanced and comprehensive understanding of the treatment and management of bilateral cleft lip and palate. Such an approach not only illuminates the surgical outcomes but also provides a holistic view of the patient journey, the potential impact of genetic factors, and the broader context of treatment. This inclusive analysis significantly benefits patients, families, and healthcare providers by deepening the understanding of this multifaceted condition and its management, supported by the foundational work of researchers whose contributions to the field offer valuable insights into the complexities of treating bilateral cleft lip and palate.

Notably, our findings underscore the significant correlation between maternal education level and awareness of prenatal care, particularly regarding vitamin intake and its impact on cleft palate risk. Enhanced education and awareness among expectant mothers, especially those under stress, could potentially reduce the incidence of cleft palate. Furthermore, through extensive epidemiological studies examining various risk factors across different populations, alongside the development of comprehensive and diverse rehabilitation protocols in areas like physiotherapy, the scientific community can make strides towards a more holistic understanding and management of cleft palate. This approach aims to provide individuals affected with a complete and optimized pathway from prevention to rehabilitation, ultimately improving their quality of life and facilitating better societal integration”.

This revised conclusion ties in the role of maternal education and awareness in relation to cleft palate, emphasizing the importance of preventive measures and comprehensive management strategies.

## Figures and Tables

**Table 1:** Gene where responsible for isolated cleft lip and/or palate.

Name of Gene	Symbol
Transforming growth factor—alpha	TGFA
Transforming growth factor—133	TGF 133
Methylene tetrahydrofolate Reductase	MTHFR
-	-
Endothelin—1	ET1
BCL3 Transcription Coactivator	BCL3
Retinoic acid receptor alpha	RARA
MSX1-Msh Homeobox 1	MSX-1

**Table 2:** Syndromes in which cleft lip and/or palate appear.

Syndromes	Name of Gene	Symbol
Waardenburg syndrome, type II A	Microphthalmia—Associated Transcription Factor	MITF
Di George syndrome	Di George syndrome chromosome region	CATCH 22
Treacher Collins syndrome mandibulofacial dysostosis	Treacle Ribosome Biogenesis Factor 1 RNA Polymerase I and III Subunit C RNA Polymerase I and III Subunit D RNA Polymerase I Subunit B	TCOF1, POLR1C, POLR1D, POLR1B
Van der Woude syndrome	Interferon Regulatory Factor—6	IRF 6
CLP-Ectodermal dysplasia syndrome	Poliovirus receptor related-1	PVRL1
Ectrodactyly, ectodermal dysplasia orofacial cleft syndrome Pigment Anomaly-Ectrodactyly Hypodontia Syndrome	Tumor Protein P63	TP63
Zollinger syndrome-3, Zellweger Syndrome	Peroxisomal Biogenesis Factor 2	(PXMP3) PEX2
Diastrophic dysplasia	Diastrophic dysplasia sulfate transporter	DTDST
Gorlin syndrome (Basal cell nevus syndrome 1)	Patched 1	PTCH1

**Table 3:** Tertiary classification of cleft cases and corresponding LASHSAL coding, where L (or l) denotes lip, A (or a) denotes alveolus, H (or h) denotes hard palate, S (or s) denotes soft palate, and U denotes uvula. Capital letters describe the cases where the anatomic feature was completely clefted, while lowercase letters describe cases with partial clefting. Values denote the number of cases (children). The last column score represents.

	Midline or Unilateral Incomplete	Unilateral Complete	Bilateral	Score
l	1	.	.	0
L	3	.	.	0
S	6	.	.	0
al	1	.	.	0.333
LA	1	.	.	0.333
sh	1	.	.	0.333
SL	1	.	.	0.333
SU	1	.	.	0.333
HSH	4	.	.	0.333
Lal	1	.	.	0.333
laHS	.	2	.	1
LAHS	.	15	.	1
Shal	.	1	.	1
SHAL	.	24	.	1
HSHAl	.	.	1	1.333
HSHAL	.	.	2	1.333
LAHSH	.	.	2	1.333
LAHSHL	.	.	1	1.667
lHSHAL	.	.	1	1.667
lahSHAL	.	.	1	2
LAHSHal	.	.	1	2
LAHSHAL	.	.	11	2

**Table 4:** Estimates of the generalized ordinal logit model.

	Odds Ratio	97.5%	2.5%	Estimate	Std. Error	z Value	Pr (>|z|)
(Intercept): 1	5.3849	0.7187	0.0480	−1.6836	0.6905	−2.4383	0.0148
(Intercept): 2	0.4718	7.7150	0.5822	0.7511	0.6592	1.1395	0.2545
Low or very low birth weight	0.9747	3.7668	0.2794	0.0256	0.6636	0.0385	0.9693
Child was born fourth or more in a row	1.3162	2.5587	0.2256	−0.2748	0.6195	−0.4435	0.6574
Men (boy)	0.8755	2.9256	0.4459	0.1329	0.4799	0.2770	0.7818
Cleft in any parent’s history	6.6475	1.2151	0.0186	−1.8942	1.0659	−1.7772	0.0755
Mother’s secondary or high education	0.6102	5.9077	0.4546	0.4940	0.6542	0.7551	0.4502
Toxic risk at mother’s work:1	4.9688	1.9260	0.0210	−1.6032	1.1524	−1.3912	0.1642
Toxic risk at mother’s work: 2	0.1344	80.8591	0.6845	2.0068	1.2173	1.6486	0.0992
Mother’s Infections or drug toxicity during pregnancy	1.1702	2.5310	0.2885	−0.1572	0.5540	−0.2838	0.7766
Mother’s stress during pregnancy: 1	0.8226	8.6182	0.1715	0.1953	0.9993	0.1955	0.8450
Mother’s stress during pregnancy: 2	9.3879	0.7837	0.0145	−2.2394	1.0182	−2.1994	0.0278
Interaction between cleft in any parent’s history and Mother’s secondary or high education	0.0370	322.1006	2.2732	3.2980	1.2637	2.6098	0.0091

**Table 5:** Estimates of the standard linear model.

	Estimate	2.5%	97.5%	Std. Error	t Value	Pr (>|t|)
(Intercept)	1.0946	0.7251	1.4641	0.1854	5.9050	0.0000
Low or very low birth weight	0.0635	−0.3011	0.4280	0.1829	0.3470	0.7296
Child was born fourth or more in a row	−0.0163	−0.3688	0.3362	0.1768	−0.0921	0.9269
Men (boy)	0.0013	−0.2641	0.2668	0.1332	0.0100	0.9921
Cleft in any parent’s history	0.2718	−0.2709	0.8146	0.2723	0.9984	0.3214
Mother’s secondary or high education	−0.1350	−0.4982	0.2281	0.1822	−0.7413	0.4609
Toxic risk in mother’s work	−0.0333	−0.4055	0.3389	0.1867	−0.1784	0.8589
Mother’s Infections or poisoning during pregnancy	0.0812	−0.2370	0.3993	0.1596	0.5088	0.6125
Mother’s stress during pregnancy	0.4454	−0.0770	0.9678	0.2620	1.6996	0.0935
Interaction between cleft in any parent’s history and Mother’s secondary or high education	−0.6610	−1.3124	−0.0095	0.3268	−2.0227	0.0468

## Data Availability

The data presented in this study are available in the article.
